# Durability of Recycled Concrete Aggregates Prepared with Mechanochemical and Thermal Treatment

**DOI:** 10.3390/ma15165792

**Published:** 2022-08-22

**Authors:** Mujaheed Yunusa, Xiaoshan Zhang, Peiqiang Cui, Xiaowu Tian

**Affiliations:** 1 State Key Laboratory of Silicate Materials for Architectures, Wuhan University of Technology, Wuhan 430070, China; 2 China Ge Zhou Ba Grp Co., Ltd., Wuhan 430079, China

**Keywords:** concrete demolition waste, recycled concrete aggregates, mechanochemical treatment, thermal treatment, asphalt pavement

## Abstract

Recycled concrete aggregates (RCAs) have low quality when compared with natural or conventional aggregates as the paste adhering to it is the key aspect that affects its functionality. Since the adhering cementitious paste weakens the adhesion between the aggregate and the binder, it becomes a decisive factor in the mechanical behavior of the asphalt mixture. It turns out that enhancing the surface of the aggregate or eliminating the paste attached to the NA (natural aggregate) is crucial for improving interfacial bonding. Therefore, the treatment and evaluation of the RCAs by laboratory testing method were studied in this research to assess their reuse in the asphalt pavement surface coarse layer. With the various techniques for removing paste from the RCA, a combination of three enhancement processes were developed for the effective removal of the cement paste, which are mechanical, chemical, and 400 °C conditioning thermal treatment. The RCAs were first charged in a Los Angeles machine for the mechanical treatment to remove parts of the attached cement. Then they were soaked in two types of acids, namely hydrochloric and sulfuric, with different concentrations to determine the most effective or optimum molarity for about a 48 h soaking duration. Then a 2 h thermal treatment was conducted on the RCA samples. After all the treatments were done, the RCA aggregates were subjected to different types of tests to examine their properties in order to ensure their full potential in terms of their physical, chemical, mineralogical, and surface microstructure characteristics. Based on the experiment design, the study intends to examine the quality of the treated recycled aggregates generated by the combination approach as well as to investigate the optimal acid concentration and type. The results show that the proposed mechanochemical and thermal treatment reinforced the quality of the RCAs when compared to the non-treated samples. Meanwhile, HCl proved to be the optimum compared to H_2_SO_4_ in most examinations of the properties. In the end, better chemical qualities were validated, and the RCA qualities were improved.

## 1. Introduction

With the rise of solid waste from construction and human activities as well as the global population increase, getting rid of waste materials is a great concern and is continuously attracting more and more attention across the world. The reprocessing of solid waste material is a central subject from the point of both sustainability and the economy. Sand, gravel, or crushed stone (natural aggregate) are inert granular materials used to fabricate concrete or asphalt. The main sources of the natural aggregates (NAs) and sand are usually pits, rivers, lakes, seabeds, and so on. Presently, aggregate production is becoming greater as it estimates to be around 20 billion tons, which causes a decrease or distortion to natural resources as well as environmental degradation and energy consumption [1]. Zongwu et al. [2] reported that the vast number of infrastructure projects are currently putting strain on the supply of natural raw resources. Management of old concrete is a major challenge for society in accordance with the reduce, reuse, and recycle concept. Shortage of space and natural resource redundancies are issues for long-term solutions for both collection and storage of concrete in large piles [3]. Hence, from different angles worldwide, research is being conducted on reuse aggregates. Rui et al. [4] investigated the reuse of red mud and steel slag to synthesize zeolite for the reduction of VOCs in asphalt. The reused or recycled aggregate generated from concrete or asphalt is also considered a resource with additional high value as an option for the natural aggregate resources, and it can also help achieve environmental preservation [5].

Many studies have been performed in line with the use of recycled concrete aggregate (RCA) from construction demolition west (CDW) as aggregate in hot-mix asphalt (HMA) [6,7,8,9,10,11,12,13,14,15,16]. Studies show that the attached cement paste on the surface of the RCAs plays a vital role in aggregate properties both physical and chemical, making the RCA have a higher porosity and less dense when compared to the NAs, which appear to be one of the main reasons for the poor quality of the RCAs than the NAs [8,17,18]. Many techniques have been applied for the effective removal of the attached cement paste from the original NAs, as the quality of the RCAs is determined by how much adhering mortar is removed. As concrete structures are aging, demolition takes place that generates concrete waste and can be recycled in treatment plants [19]. To enhance the RCA, major approaches have been found, including the removal of paste adhered to the RCA via chemical treatments [20,21,22,23,24], thermal treatments (traditional heating, microwave heating) [3,25], mechanical treatments [26], the combination of heating and mechanical treatments [27], and the combination of chemical and mechanical treatments [28,29]. Results show that sulfuric and hydrochloric acids proved to be excellent for the removal of cement paste from aggregate surfaces [7].

Many techniques have been applied for effective removal of attached cement paste from the original NAs, as the quality of the RCAs is determined by how much adhering mortar is removed. Most methods applied currently are (i) chemical treatment (acid treatment), in which samples of the RCAs are immersed in a solution of acids such as HNO_3_, H_2_SO_4_, HCl, CH_3_COOH, and so on [9,23,24]; (ii) Heat treatment, in which the RCAs are charged into a furnace for the thermal treatment and soak in water immediately [30]; (iii) Mechanical treatment, in which the RCAs are processed in a ball milling or jaw crusher [31]. These approaches, however, enhance the RCAs in both their physical and chemical properties. Tam et al. [9] presoaked the RCA in HCl, H_2_SO_4_, and H_3_PO_4_ solutions to dissolve the old mortar adhering to the RCA surface, and then they examined the fundamental parameters of the new RCA. The amount of acid solution used needs to be kept within a reasonable range to remove the old mortar efficiently and produce a high-quality RCA. The effects of various acid concentrations (0.1, 0.5, and 0.8 mol of HCl) and treatment times on the characteristics of aggregate and concrete were evaluated by Guneyisi et al. [32]. They discovered that the time spent immersed in an acid bath had no discernible effect on the quantity of mortar removed and that the application of a low concentration of HCl at 0.1 molarity might potentially remove the loose adherent mortar on the RCA surface. RCAs are becoming popular as implementation processes for production are taking place in Latin America, in countries such as Brazil and Mexico. Yet the present-day technology has challenges of high absorption ratio as well as low density, making them impossible to perform to their full potentiality [33]. The remains of the cement paste on aggregate surface is the reason why low quality is observed, as absorption ratio and specific gravity are negatively affected [25,34]. Hence, many studies have been conducted to check measures for effective removal of attached cement paste from aggregates, and the majority of research involved the use of mechanical means such as crushers of any type or grinding the waste concrete to remove the attached cement paste, or the use of acid or heat treatment as beneficiation processes. While a crushing process, for instance, can result in a greater reduction of attached cement paste content, as it involves the crushing of weaker aggregates to fine aggregates, the increase in cost is becoming a great concern, as is the overall reduction in the production capacity of coarse aggregates [30,35].

The goal of the treatment is to improve the quality of RCAs. Therefore, the treated RCAs have a better compressive strength of fabricated concrete than the untreated RCAs. Thus, several kinds of treatment for the RCAs appear to be positively effective, especially when the RCAs are partially replaced, and when the performance of the mixtures with the RCAs are enhanced [36,37,38,39]. From a physical properties point of consideration, the RCAs increase water absorption, chloride ion penetration, and so on [29,40]. The aggregate production in the world is becoming so high as it hits a billion tons, and this shows that development projects are starting to appear around the world [41]. Subsequently, the shortage of space for used aggregate disposal will be very challenging as the aggregates reach an extent of a billion tons annually. Therefore, recycling C&D waste will be the most interesting topic due to environmental concerns, and it will be available and less expensive [42,43]. With the level of technology and development waste, materials are viewed as resources or by-products that, after processing, can be used for a variety of purposes. In a laboratory conducting research on crushed concrete aggregate, the researchers emphasized that any findings on demolition waste should only be related or applied to materials of the same types and not to other recycled materials, as many different types are produced [44].

## 2. Objectives of This Research

This article aims to assess the efficacy of the combined enhancement methods for, RCAs, such as mechanical (Los Angeles abrasion), chemical (acids), and thermal treatments, and to analyze both the physical and chemical properties of the RCAs after the treatment. The favorable parameter of the mechanochemical and thermal treatment was determined and considered for asphalt pavement. The results of this work will be interesting mostly in countries where a shortage of natural aggregate is being experienced.

## 3. Materials and Methods

### 3.1. Materials

**Figure 1:**
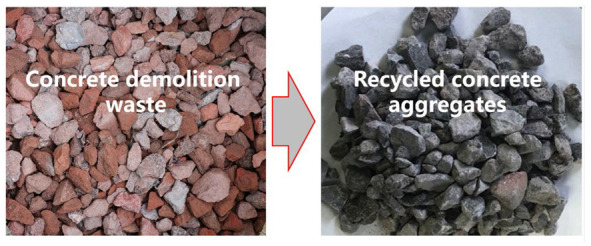
The recycled concrete aggregates were selected from the concrete demolition waste.

The RCAs materials used were obtained from recycling sites in Hebei province, during the reconstruction of city villages as shown in Figure 1. They were cracked with a movable aggregate crusher. Table 1 presents the basic properties of the used RCAs. Hydrochloric acid (HCl) and sulfuric acid (H_2_SO_4_) were used throughout this research for the chemical treatment, as sulfuric and hydrochloric acids are effective for cement paste removal. The chemical treatment approach of pre-soaking the RCA in acidic solutions has shown to be a useful way of removing adhering mortar and improving the characteristics of the RCA [45]. It involves an acid attack caused by the interaction between an acid (HX) and the calcium hydroxide (CH) component of the cement paste. This reaction results in the production of a highly soluble calcium salt (CX), where X is the acid’s negative ion, which is then readily removed from the cement paste, weakening its overall structure (Equation (1)) [46,47].

HX + CH → CX + H(1)

Granite, feldspar, quartz, and mica can be easily dissolved in strong acids such as hydrofluoric acid [48,49].

### 3.2. Experimental Program

**Figure 2:**
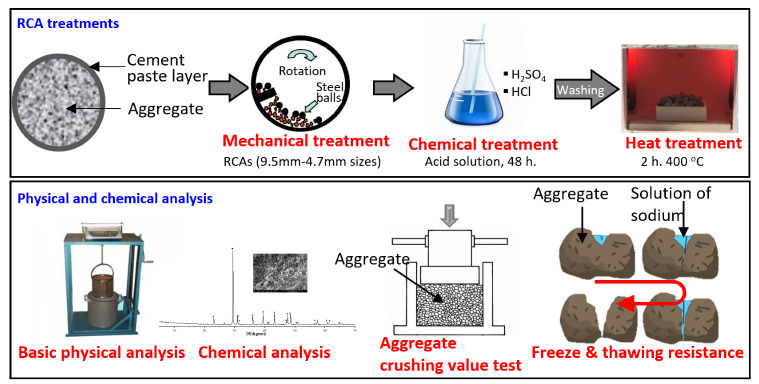
Treatment processes and test programs of this study.

Figure 2 shows the approaching treatment steps and tests performed for characterizing the effect of the mechanical, chemical, and thermal treatment on the RCAs. It includes the basic conditions of the mechanical treatment, the chemical treatment, and the thermal treatment that were used in this study. Laboratory tests, including chemical analysis, crushing value test, freeze, and thawing resistance test, were then undertaken on the treated RCAs.

#### 3.2.1. Mechanical Treatment

In the first stage, the mechanical treatment was conducted on the reused RCAs with a Los Angeles abrasion machine to disintegrate part of the mortar from the aggregate. The crushed concrete aggregate, which was less than a grain size of 20 mm, was sieved and separated from the fine and dust fractions. The Los Angeles machine was used mainly for the mechanical treatment, as it was significant for indicating the relative quality or the competence of the aggregate. Meanwhile, its processing system performs a degradation from a combined action of abrasion or attrition, impact, and grinding in a rotating steel drum containing 12 spheres, which clearly shows that, at a low number of revolutions, the adhering mortar can be removed without causing severe damage to the aggregate itself. Therefore, such a principle was applied as the mechanical treatment to remove or reduce the adhering mortar, the material was separated to the designated fraction, and further treatment proceeded. The RCA samples (10 kg oven dry weight) were rubbed against each other and the steel balls were spun for 100 rotations of the spinning drum using Los Angeles abrasion testing equipment with a load of 10 steel balls [25].

#### 3.2.2. Chemical Treatment

The mechanically treated RCA samples were firstly immersed in HCl and H_2_SO_4_ water solution of four different concentrations, which were 1 M, 2 M, 4.5 M, and 5 M at ambient temperature. 1 M or molarity (M) is a unit to measure the concentration of a solution. The concentration of the acid solution is a key factor affecting the results of the treatment, intending to obtain the optimum treatment conditions [41]. The same duration of soaking, which was 48 h [41], was used for the whole sample, as can be seen in the experimental program. The amount of mortar removed was not significantly affected by the timeframe of soaking [32]. Then the RCA samples were taken out and washed for acidic solvent removal, and then dried in a ventilated place at room temperature for 24 h.

#### 3.2.3. Thermal Treatment

In this method, thermal stresses were generated through heating of the chemical-treated RCAs at a temperature of 400 °C using a glass laboratory furnace JZ-4-1300 for 2 h and allowed to cool. Thermal loading was 400 °C as the decomposition of the hydrated calcium acetate occurs at 380 °C [50]. When the demolished concrete is heated to around 300 °C, dehydration causes the cement paste to become brittle [3]. In these treatment processes, mechanical crushing is followed by chemical soaking and finally by thermal roasting of the RCAs, as can be seen in Figure 2.

## 4. Results and Discussion

### 4.1. Chemical Analysis

#### 4.1.1. X-ray Diffraction

**Figure 3:**
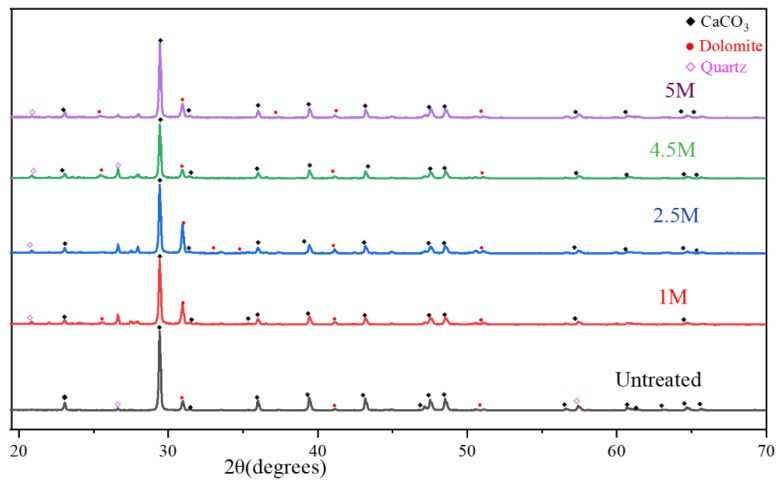
XRD pattern of samples treated with H_2_SO_4_/untreated.

**Figure 4:**
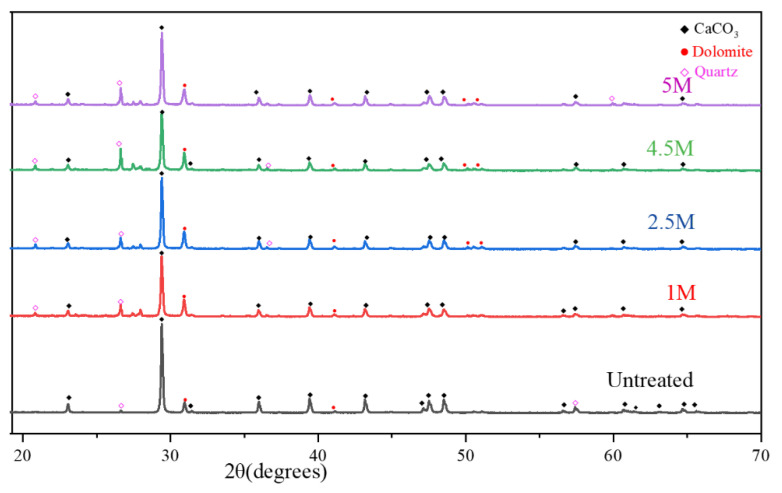
XRD pattern of samples treated with HCl/untreated.

To determine the effect of the three types of treatment proposed for the RCAs, the XRD test analysis were performed. The pattern of the untreated and various treated RCAs is shown in Figure 3 and Figure 4. The major peaks were calcium carbonate, dolomite, and quartz; meanwhile, the intensity of peaks was inversely proportional to acid concentration, and less intense peaks disappeared with molarity variation. The intensity of compounds was reduced more with HCl acid. The minor peaks were difficult to identify due to the high intensity of the above-mentioned minerals. Calcium oxide was present in the form of calcite, which shows that the cement paste was highly carbonated.

#### 4.1.2. X-ray Fluorescence

**Figure 5:**
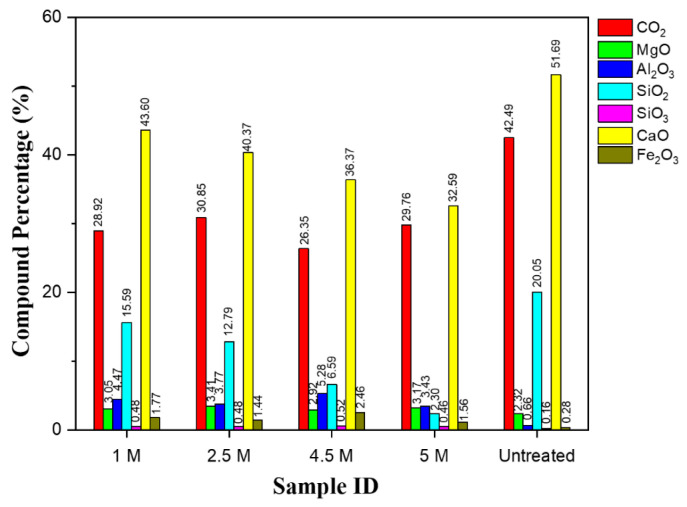
Compound composition of samples treated with H_2_SO_4_/untreated.

**Figure 6:**
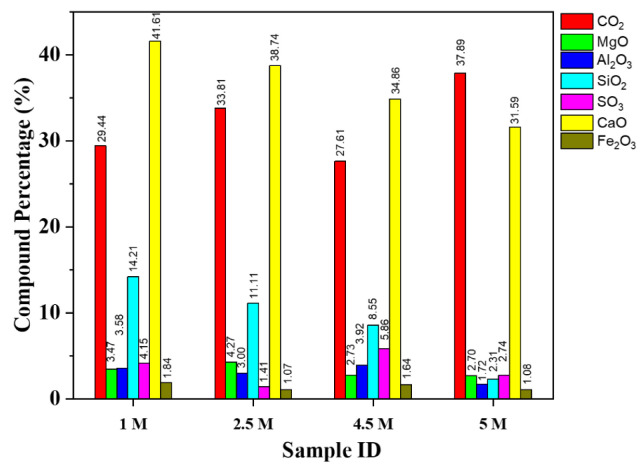
Compound composition of sample treated with HCl.

Chemical analysis by XRF was carried out on the untreated and treated RCA samples as presented in Figure 5 and Figure 6. Peide et al. [51] also used XRF to detect the chemical composition of basalt and steel slag aggregate. It can be observed in Figure 5 that the compound composition of the untreated RCAs contains a considerable number of cementitious elements, as do the treated samples. Cementitious minerals or compounds fluctuate in quantity with respect to acid concentration, but the main concern compounds of silica (SiO_2_) and quicklime (CaO) decrease in amount. Carbon dioxide (CO_2_) contents were increased significantly, and this was attributed to the formation of CaCO_3_ under chemico-thermal treatment, which agrees with prior work reported in the literature [40]. A drastic change was observed with the treated HCl RCA samples. Silica contents were a concern as they determine the affinity of the coarse aggregates to the bitumen binders, as a good coating and adhesion of bitumen to the aggregate is essential. Generally, aggregates with high silica contents (acid) have a good affinity for water (hydrophilic) but not for bitumen [52,53]. They are more likely to suffer from stripping after exposure to water than those with lower silica contents (basic) because they have an affinity for bitumen rather than water. Consequently, a loss of the bond between the aggregate and the bitumen in service due to stripping has a major detrimental effect on the integrity of an asphalt pavement. Therefore, this analysis gives us an awareness of the cementitious compound contents as well as the silica contents.

#### 4.1.3. Surface Microstructure

**Figure 7:**
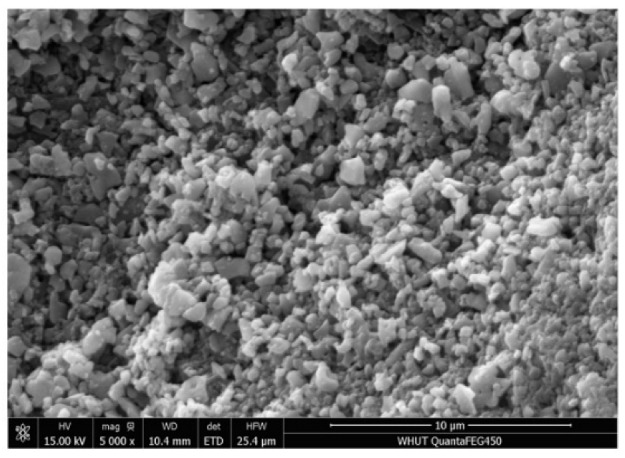
SEM (SE mode) Surface microstructure of the untreated sample.

**Figure 8:**
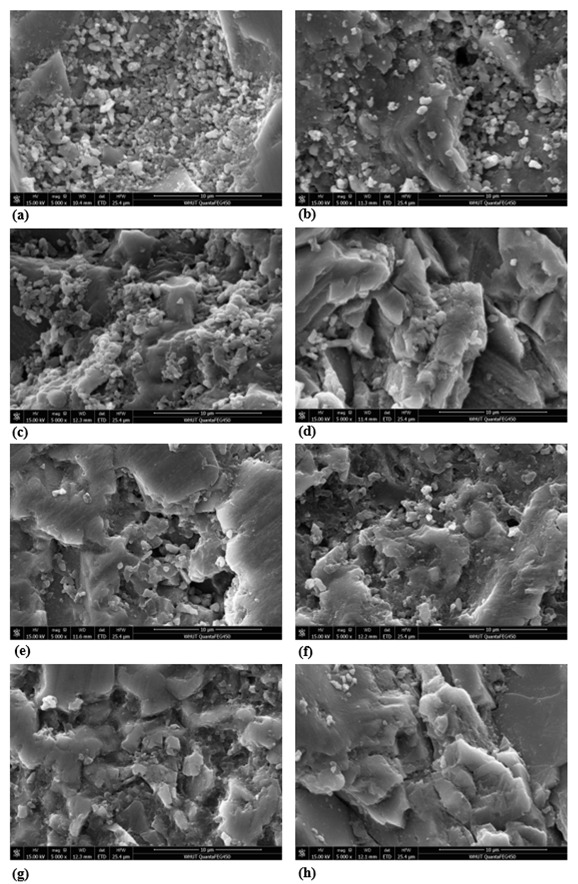
SEM (SE mode) surface microstructure of various samples, (**a**) 1 M H_2_SO_4_, (**b**) 2.5 M H_2_SO_4_, (**c**) 4.5 M H_2_SO_4,_ (**d**) 5 M H_2_SO_4_, (**e**) 1 M HCl, (**f**) 2.5 M HCl, (**g**) 4.5 M HCl, (**h**) 5 M HCl.

Different methods were used for the aggregate characterization. Wang et al. [54] characterized the aggregate morphologies based on a three-dimensional curvature analysis. The microstructure of the various untreated and treated samples was identified with the help of SEM. It can be seen from the SEM image that the samples are porous, which mainly relies on the presence of cement pasts and the formation of chemical compounds during the chemical treatment, whereas the sulfuric acid treatment causes much greater porosity. This agrees with the compound composition reported in XRF from Figure 5, but it applies less to the loose particles as those particles are removed by the acids relative to their concentration. As revealed in Figure 7, the surface of the untreated RCAs was associated with a considerable amount of porosity, which was created by the size reduction process from the primary crushing, but it was highly covered by the loosely bonded cement mortar because the chemical treatment that removes the loose particles was absent. As shown in Figure 8a–h, the surface of the RCAs samples was treated with 1 M, 2.5 M, 4.5 M, and 5 M sulfuric acid and hydrochloric acid, respectively. Acid treatment with HCl at low molarities (0.1 M and 0.5 M) in the RCA, according to Ismail and Ramli [21,55], lowers the quantity of weakly attached particles, producing a cleaner, smoother surface with less-sharp angles [56]. The authors also discovered that when the molar concentration increases, the brittle particles of the mortar are liberated as a result of the increasing attack on the mortar induced by the increased addition of H+. It can be observed that treated samples are denser and smoother when compared to untreated samples.

### 4.2. Physical Comparison Analysis

#### 4.2.1. Basic Mechanic Properties

The effect of the concentration treatment of the two types of acids, which was one of the key factors in this investigation, can be observed in Table 2, which shows the physical properties of the treated and untreated RCA samples. ASTM C127 detailed description was used for the test. The primary parameters used to categorize the RCA quality are water absorption and dry density, which played a vital role [50]. Overall, the lowest water absorption was achieved by using 2.25 M H_2_SO_4_. However, a further reduction could not be achieved by using a higher concentration of H_2_SO_4_, as observed. This shows that the water absorption of the treated RCAs with the two types of acids had a significant variation with respect to the concentration. Water absorption was decreased after the RCA was superficially treated in modest amounts of acidic solutions [9]. All samples treated with HCl had a lower absorption ratio than the untreated samples. Purushothaman et al. [57] discovered that the RCA treated with HCl and H_2_SO_4_ in five particle sizes (20.0, 16.0, 12.5, 10.0, and 4.75 mm) at a concentration of 0.1 M and pre-soaked for 24 h exhibited a 41% and 58% decrease in water absorption for HCl and H_2_SO_4_, respectively, compared to the untreated RCA. Such a result can be attributed to more hydration products and possibly some NAs being dissolved by the acid at higher concentrations, confirming that the fine powder attached to the coarse aggregate was washed and the cement paste was removed by the chemical reaction. Sulfuric and calcium hydroxide in the cement paste induced the production of gypsum dehydrate, as can be seen in Equation (2), and the removal of calcium hydroxide from the aggregate surface. On the other hand, a reaction between hydrochloric acids and calcium hydroxide in the cement paste resulted from the production of water and calcium chloride as seen in Equation (3). As a result, more pores were produced in the treated RCA samples, leading to higher water absorption. It can be understood that the density, apparent density, and apparent relative density of all the treated samples were higher than that of the untreated ones, with respect to concentration increase. However, after acid immersion, the physical characteristics of the RCA increased, with a bigger improvement for the 10 mm aggregates (4.7%) than for the 20 mm aggregates (2.2%), owing to the amount of adhering mortar being higher in the smaller aggregates than in the coarser aggregates [30]. Increases in RCA density resulted in a considerable decrease in RCA water absorption because of the relationship between aggregate density and absorption [56,58,59]. With increasing acid concentrations, the density of the RCA rose. Al-Bayati [60] also observed improvements in the bulk density for C_2_H_4_O_2_ and HCl of 7.37% and 5.40%, respectively, when compared to the untreated RCA samples. Saravanakumar et al. [24] discovered that following the RCA treatment with three acids (H_2_SO_4_, HNO_3_, and HCl), the bulk density varied by less than 10%, 13%, and 13%, respectively, as compared to the untreated RCA samples, which varied by 15% in comparison to the NA. Some loose part of the RCAs was removed by the acid treatment.

H_2_SO_4_ + Ca(OH)_2_ → CaSO_4_·H_2_O (2)

HCl + Ca(OH)_2_ → CaCl_2_·H_2_O (3)

#### 4.2.2. Freezing and Thawing

The stability of the RCAs is an important physical property, as it is changed by different acids as well as their corresponding molarity variation ranging from 1 M to 5 M. This test is of great significance as it produces information that is vital in understanding the soundness and durability of aggregates being subjected to freezing and thawing. Aggregates with water absorption values of 2% or less are considered a suitably freezing and thawing action without further testing, while aggregates with greater than 2% can be very resistant. But for assurances and confirmation, a direct test should be performed for these properties. This process covers the testing of aggregates to determine resistance to disintegration by a repeated freezing and thawing cycle using the Test Method LS-614. To compute this phenomenon, calculate the percent loss and weight for each sample fraction based on the material retained on the sieved size used as follow:

(4) $$\mathrm{Percent}\;\mathrm{Loss}=\frac{Original\;Mass-Mass\;Retain\;after\;test\;}{Original\;Mass}\times 100$$

The loss should be nearest to 0.1%. Therefore, the higher the percentage loss the lower the resistance to freezing and thawing.

**Figure 9:**
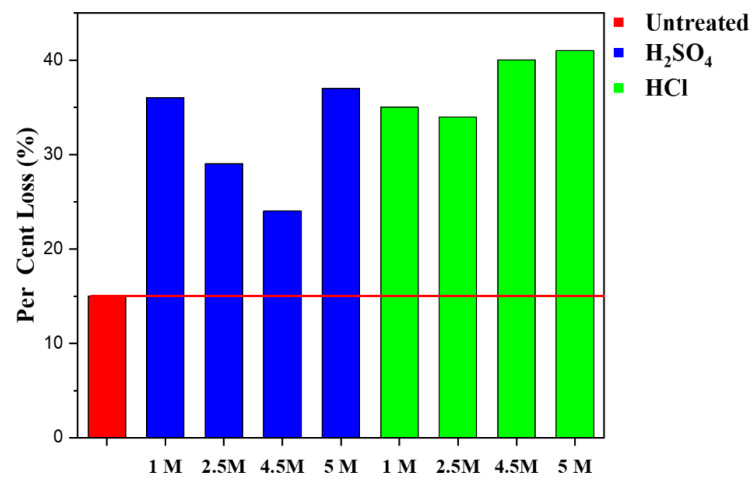
Freezing and thawing of untreated and various treated RCA samples at different concentrations.

The highest percentage loss in the RCA samples was for 5 M HCl treated samples, followed by 4.5 M HCl, as can be observed in Figure 9. It can be inferred that 2.5 M proved to be the most stable out of all the HCl treated samples. It shows that stability is not solely proportional to concentration increment, which is also confirmed with the H_2_SO_4_ treated samples, as 4.5 M is the most stable with the least percentage lost, followed by 2.5 M, 1 M then 5 M. The treated RCA samples are less stable because they all have a higher loss percentage than the untreated samples, and this shows that the higher the percentage the lower the resistance to freezing and thawing. It can be inferred that for each category of aggregate, the techniques used for the freezing and thawing test contribute to the reaction between acids and sodium chlorides, although other treatment types such as the mechanical and the thermal may affect this slightly, which makes the trends of stability or percentage subject to fluctuation. Reduced adhering mortar content has been shown to improve the RAC’s freeze-thaw resistance [61]. Nevertheless, the stability differences between the untreated RCAs and the treated samples can be negligible as the negative effect has not outweighed the freezing and thawing standard, and such a situation is a result of the method used for the assessment.

#### 4.2.3. ACV of Recycled RCAs

**Figure 10:**
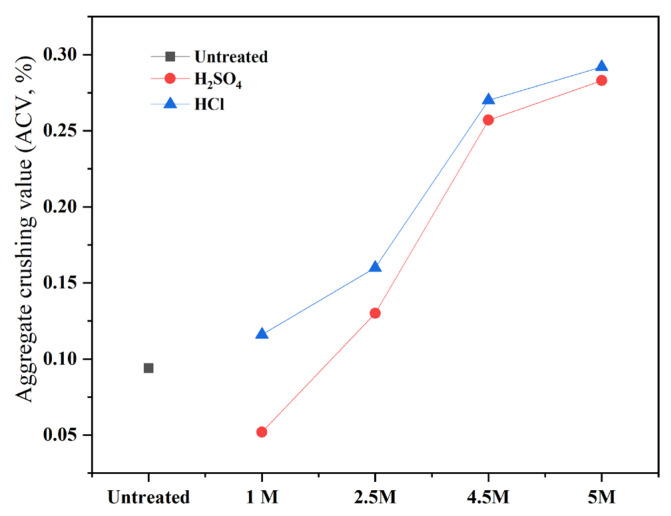
Aggregate crushing value of various RCA samples.

The aggregate crushing value (ACV) obtained for the corresponding RCAs test with a series of aggregates treated with acids in comparison to the control sample is shown in Figure 10. A few trends can be observed with the acid treated RCAs, as the aggregate crushing value increases to a greater extent in proportion to the concentration increase for the two types of acids. However, the aggregate immersed in sulfuric acids has a lower aggregate crushing value when compared to the aggregate immersed in a hydrochloric acid water solution, which agrees with the investigation reported in [41]. An explanation for these tendencies is that immersing recycled fine aggregates in a sulfuric acid solution leads to the production of gypsum in a reaction as shown in Equation (3), and if the gypsum on the washed aggregate surface is not completely removed, it will come into contact with the calcium alumina of the cement and generate ettringite as shown in Equation (4), thereby giving rise to more voids [35,40].

Ca(OH)_2_ + H_2_SO_4_ → CaSO_4_ + 2H_2_O (5)

C_3_A·CaSO_4_·18H_2_O + 2CaO·2H_2_O + 2SO_3_ + 12H_2_O → C_3_A·3CaSO_4_·32H_2_O (6)

It can be stated that the treatment of the RCAs positively influenced the aggregate crushing value, particularly samples treated with HCl, as all values have a higher ACV than the other samples.

## 5. Conclusions

A methodology for producing high-quality recycled aggregate using mechanical, chemical, and thermal procedures has been developed to encourage the usage of RCA. Following a discussion of the technology, a future scenario of an aggregate recycling system is examined in this research to assess the technology’s applicability using an optimization model. According to the results, the proposed three methods carried out in this study improved the quality of the RCAs to a great extent. Assessment of the quality improvement was confirmed by laboratory evaluation of the physical and chemical properties of the RCAs. According to the discussed results, the following conclusions are drawn:

1. The mechanism behind this mechanochemical and thermal treatment is that high-quality RCAs were obtained; these were characterized by a low absorption ratio, a high density, and a better aggregate crushing value compared to the untreated and conventional aggregate. Because of the inferred low amount or complete absence of clay content, swelling, and moisture sensitivity, the stripping phenomena was therefore minimized. The findings lead to the conclusion that treatments have the potential to improve the quality assessment of the RCA.
2. The treated RCA samples have superior chemical properties compared to the conventional and untreated, which were identified by XRF, XRD, and SEM with respect to concentration variation, compound composition, compound intensity, and microstructure, which were positively influenced as they have a significant impact on the aggregate, which determines asphalt performance as well.
3. The cementitious compounds and silica contents of the treated RCAs are reduced when compared to the untreated samples, with an increase in the concentration in H_2_SO_4_ and HCl as well. Meanwhile, other compounds’ variation is not significant, and changes are not directly proportional to molarity increase. The amount of adhering mortar removed is not directly related to the amount of time immersed in the acidic solution. This occurs as a result of the sulfate ions (SO_4_^2−^) being consumed in the first stages of the process during the removal of mortar. When the acidic concentrations are raised to levels greater than 3 M, the paste that was present in the RCA is completely removed [20].
4. However, the soundness was assessed by subjecting the RCA samples to freezing and thawing cycles. The treated RCA samples have better durability properties, and H_2_SO_4_ proved to be better than HCl, although the difference is insignificant, as the chlorine element concentration is increased due to NaCl recrystallization. Therefore, if other assessment techniques are used, HCl performs better as it has proven in other tests as well as in the literature.

## Figures and Tables

**Table 1:** Basic properties of the recycled concrete aggregates.

Properties		Values
Physical properties	Water absorption %	3.199
	Density (g/cm^3^)	2.65
Chemical properties, composition (%)	Quartz	NA
	Calcium Oxide	51.69
	Iron Oxide	0.28
	Aluminum Oxide	0.66
	Sulfur Trioxide	0.16
	Sodium Oxide	0.193
	Potassium Oxide	0.275

**Table 2:** Effect of treatment conditions on basic mechanic properties of RCAs.

Properties of RCA	Untreated RCA	Treated RCAs (Acid Concentration and Thermal Treatment at 400 °C for 2 h)							
		HCl Acid				H_2_SO_4_ Acid			
		1 M	2.5 M	4.5 M	5 M	1 M	2.5 M	4.5 M	5 M
**Water absorption %**	3.199	2.48	2.89	3.12	2.734	4.027	2.291	4.20	3.591
**Density (g/cm^3^)**	2.65	2.67	2.716	2.836	2.86	2.668	2.706	2.896	2.905
**Apparent density (g/cm^3^)**	2.72	2.752	2.759	2.759	2.759	2.753	2.76	2.781	2.804
**Apparent relative density (g/cm^3^)**	2.687	2.792	2.782	2.767	2.752	2.851	2.924	2.939	2.954
